# The assessment of family caregivers’ anxiety in pediatric epilepsy : a cross-sectional sudy

**DOI:** 10.1192/j.eurpsy.2022.996

**Published:** 2022-09-01

**Authors:** A. Zouari, N. Charfi, A. Guermazi, M. Mnif, W. Bouchaala, S. Ben Ncir, F. Kammoun, M. Maalej, C. Triki

**Affiliations:** 1 Hedi Chaker Hospital, Psychiatry C, sfax, Tunisia; 2 Hedi Chaker Hospital, Pediatric Neurology, sfax, Tunisia; 3 Faculty of medicine of Sfax, Lr19es15 Pediatric Neurology, sfax, Tunisia

**Keywords:** Anxiety, epilepsy, Caregiver, Pediatric

## Abstract

**Introduction:**

Pediatric epilepsy is a debilitating disease that impacts not only children with epilpsy but also persons arround them. It is often considered as a source of anxiety for family caregivers.

**Objectives:**

Assess the level of anxiety in caregivers of children with epilepsy and to identify factors related to it.

**Methods:**

We conducted a cross-sectional, descriptive and analytical study between July and October 2020. It included caregivers of children with epilepsy hospitalized in the pediatric neurology department of Sfax. We used the STAI-Y scale to assess the level of state anxiety (STAI-AE).

**Results:**

Forty four womens participated in our study. Low socio-economic level was found in 31.8% of cases. The average age of children was 4.9 years. The mean duration of epilepsy was 2.2 years. It was comorbid with an autism spectrum disorder or an intellectual disability in 15.9% of cases. In 70.5% of the cases, the children were dependent on their caregivers in their daily lives. The level of anxiety was moderate in 27.3% and high to very high in 13.6% of them. A higher state-anxiety score was correlated with a longer duration of epilepsy (p=0.033), a lower familial socioeconomic level (p=0.013) and a higher number of children in family (p=0.048).

**Conclusions:**

Pediatric epilepsy is associated with significant level of anxiety in family caregivers. This anxiety increases with the duration of the disease and with the presence of socioeconomic and family difficulties. Thus, psychosocial support for caregivers should be integrated into a global approach of the disease.

**Disclosure:**

No significant relationships.

